# Effect of Acute Gamma Irradiation on *Curcuma alismatifolia* Varieties and Detection of DNA Polymorphism through SSR Marker

**DOI:** 10.1155/2014/631813

**Published:** 2014-02-25

**Authors:** Sima Taheri, Thohirah Lee Abdullah, Zaiton Ahmad, Nur Ashikin Psyquay Abdullah

**Affiliations:** ^1^Department of Crop Science, Faculty of Agriculture, University Putra Malaysia (UPM), 43400 Serdang, Selangor, Malaysia; ^2^Agrotechnology and Biosciences Division, Malaysian Nuclear Agency (Nuclear Malaysia), 43000 Bangi, Selangor, Malaysia

## Abstract

The effects of eight different doses (0, 10, 20, 25, 35, 40, 60, and 100 Gy) of acute gamma irradiation on 44 (three varieties of *Curcuma alismatifolia*: Chiang Mai Red, Sweet Pink, Kimono Pink, and one *Curcuma* hybrid (Doi Tung 554) individual plants were investigated. Radiation sensitivity tests revealed that the LD_50_ values of the varieties were achieved at 21 Gy for Chiang Mai Red, 23 Gy for Sweet Pink, 25 Gy for Kimono Pink, and 28 Gy for Doi Tung 554. From the analysis of variance (ANOVA), significant variations were observed for vegetative traits, flowering development, and rhizome characteristics among the four varieties of *Curcuma alismatifolia* and dose levels as well as the dose × variety interaction. In irradiated plants, the leaf length, leaf width, inflorescence length, the number of true flowers, the number of pink bracts, number of shoots, plant height, rhizome size, number of storage roots, and number of new rhizomes decreased significantly (*P* < 0.05) as the radiation dose increased. The cophenetic correlation coefficient (CCC) between genetic dissimilarity matrix estimated from the morphological characters and the UPGMA clustering method was *r* = 0.93, showing a proof fit. In terms of genetic variation among the acutely irradiated samples, the number of presumed alleles revealed by simple sequence repeats ranged from two to seven alleles with a mean value of 3.1, 4.5, and 5.3 alleles per locus for radiation doses of 0, 10, and 20 Gy, respectively. The average values of the effective number of alleles, Nei's gene diversity, and Shannon's information index were 2.5–3.2, 0.51–0.66, and 0.9–1.3, respectively. The constructed dendrogram grouped the entities into seven clusters. Principal component analysis (PCA) supported the clustering results. Consequently, it was concluded that irradiation with optimum doses of gamma rays efficiently induces mutations in *Curcuma alismatifolia* varieties.

## 1. Introduction

The genus *Curcuma *is a member of the Zingiberaceae family that has recently become popular for the use as flowering pot plants and cut flowers. Most *Curcuma* species are used as medicinal herbs or for culinary purposes. However, some possess aesthetic value as ornamentals such as *Curcuma alismatifolia *which is a monocotyledonous perennial, originating from the tropical and subtropical areas of northern Thailand and Cambodia. It has great potential for use as cut flowers and flowering pot plants and as a garden plant for tropical landscaping in various regions [1]. *C. alismatifolia* has flowering stems comprising of a showy inflorescence with several apical bracts on a long peduncle. Most basal bracts are green, but the distal ones, more numerous than the green ones, are purplish pink prominent elliptical bracts which determine the attractiveness of the flowering stems. Both types of bracts bear two to seven small axillary flower buds. Open flowers are small and have a purple flag petal [2]. Few breeding programs have been carried out to improve this species.

Mutation induction and selection of mutants have been powerful tools for plant breeding as well as for physiological and molecular studies for the past 80 years. X-ray, gamma ray irradiation, and chemical treatments have been used for mutation breeding in a wide range of plants [3]. Gamma rays are a type of ionizing radiation which interacts with atoms or molecules to produce free radicals in cells which damage or modify important components of plant cells and affect differently the morphology, biochemistry, and physiology of plants. Induced mutation is highly effective in enhancing natural genetic resources for the development of improved new cultivars among vegetatively propagated crops. Some important ornamental plants, for both cut flowers and potted plants that have been used in mutation breeding, are chrysanthemums [4, 5], orchids [6] roses [7], pelargoniums [8], and canna [9].

The estimation of genetic variation on the basis of morphological traits alone, which are the product of gene and environmental interactions, does not determine the actual level of genetic variation among studied individuals [10]. Several molecular markers such as random amplified polymorphic DNA (RAPD), intersimple sequence repeats (ISSR), simple sequence repeats (SSR), and amplified fragment length polymorphism (AFLP) with different advantages and disadvantages have been employed in genetic variation studies of *Curcuma* species [10–15]. RAPD markers are relatively easy to generate but may not be variable enough for some applications or may have problems with reproducibility. Among the robust class of molecular markers, microsatellites or simple sequence repeats (SSRs) are extremely powerful tools for estimating genetic variation with high reproducibility in a variety of plant species. These markers are characterized by the presence of 1–6 nucleotide repeats within the coding and noncoding regions [16, 17] of the genome which are codominant, hypervariable, and multiallelic in nature [18]. Genomic SSR markers have been developed in economically important spice crops such as *Zingiber officinale* [19], *Vanilla planifolia* [20], and *Piper nigrum* [21]. The development of 17 EST-SSR and 17 genomic SSR markers has been recently reported in turmeric (*Curcuma longa L.*) [13, 22]. Using genetic markers for internal quality control, it is possible to distinguish induced mutations from any nonmutational genetic variability and hence unequivocally demonstrate that mutations induced by gamma irradiation were the major source of genetic variability [23, 24]. This study was designed to determine the optimum dose of acute gamma radiation for selected *C*. *alismatifolia *varieties, describe the morphological variations as affected and developed from acute gamma irradiation, and elucidate the genetic variation among the irradiated *C*. *alismatifolia *varieties using microsatellite DNA markers.

## 2. Materials and Methods

### 2.1. Plant Materials

The rhizomes of three* C. alismatifolia* varieties—Chiang Mai Red (SK 2051/12), Sweet Pink (SK 2052/12), Kimono Pink (SK 2054/12), as well as one *Curcuma* hybrid, Doi Tung 554 (SK 2053/12)—were provided from the *Curcuma* Nursery (Ubonrat) in Doisaket District, Chiang Mai 50220, Thailand (Table 1).

### 2.2. Gamma Irradiation for Radiation Sensitivity Test

Irradiation of the plant materials was conducted in the Faculty of Science and Technology, University Kebangsaan Malaysia (UKM) using a Gammacell 220 Excel Irradiator (MDS Nordion, Ottawa, ON, Canada). The source of gamma rays was Cobalt 60. Prepared rhizomes in the budding stage were acutely irradiated with different doses of 10 (12.8 Sec.), 20 (25.6 Sec.), 25 (31.1 Sec.), 35 (43.6 Sec.), 40 (51.2 Sec.), 60 (80 Sec.), and 100 Gy (130 Sec.). In each variety, 20 rhizomes were treated for sensitivity testing at each dose. After irradiation, the rhizomes were planted in 25 cm pots containing growth media consisting of topsoil : cocopeat : rice husk at the ratio of 1 : 2 : 1. Radiation effect on test plants was recorded in terms of the mortality rate (%) after exposure to the gamma radiation. Number of mortal rhizomes were counted 40 days after planting (at each treatment) and expressed as percentage of the total number of rhizomes planted. The experiments were conducted in Green, house number 1, Field 2, Faculty of Agriculture, University Putra Malaysia (UPM), Malaysia. The recorded data of mortality percentage were analyzed by PoloPlus (Probit and logic analysis) software, version 2.

### 2.3. Induction of Mutation with Selected Doses of Gamma Radiation

Based on LD_50_ and obtained confidence limits of irradiation dose, 20 rhizomes from each variety were irradiated (March 2011) with gamma rays at doses of 0 (control), 10 Gy, and 20 Gy. The experiment was designed as 4 (variety) × 3 (dose) RCBD with five blocks and four replications for each sample.

### 2.4. Morphological Data

Fourteen morphological traits included vegetative traits, flowering development, and rhizome characteristics data were recorded during March 2011 to September 2011 for four varieties of *C. alismatifolia* (Table 2). The traits included number of new shoots, leaf length, leaf width, leaf number, plant height, number of days to visible bud, inflorescence length, number of days to anthesis, number of days to senescence, number of true flowers, number of pink bracts, number of rhizome, rhizome size, and number of storage roots.

## 3. SSR Analysis

### 3.1. DNA Isolation

Leaves of all mutants and control individuals were stored at −70°C until used for DNA extraction. DNA was isolated from leaves of selected 44 individuals with morphological variations using cetyltrimethylammonium bromide (CTAB) extraction buffer [25]. The extraction buffer comprised of 2% (w/v) CTAB, 1.4 mM NaCl, 100 mM Tris-HCL PH 8.0, 20 mM EDTA, 2% (w/v) PVP, and 2% (v/v) *β*-mercaptoethanol. The mixture was incubated at 65°C for 1 hour, followed by two extractions with chloroform/isoamyl alcohol (24 : 1). Isopropanol was used to precipitate nucleic acids and the pellet obtained was washed with 70% ethanol, dried, and dissolved in a Tris-EDTA (TE) buffer (10 mM Tris-HCl, pH = 8.0, and 1 mM EDTA, pH = 8.0). Coprecipitated RNA was removed by digestion with RNAse. After one hour incubation at 37°C, the concentration and purity of isolated DNA were determined using NanoDrop 2000 (Thermo Fisher Scientific Inc.) in the range of 250 to 900 ng/*μ*L which was adjusted to 70 ng/*μ*L. The quality was verified by electrophoresis on 0.8% agarose gel.

### 3.2. PCR Amplification and Product Electrophoresis

Polymerase chain reaction (PCR) was carried out for 17 SSR primers which were developed for *Curcuma longa* in previous studies [26]. PCR was carried out in a 25 *μ*L reaction volume containing 70 ng/*μ*L DNA and 2X DreamTaq Green PCR Master Mix (Fermentas, International Inc., USA) with 0.4 *μ*M primer. Amplification was performed in a thermal cycler (Bio-Rad Laboratories, Inc., USA) for a total of 40 cycles. An initial denaturation of the template DNA at 94°C for 3 minutes was followed by 10 cycles of 94°C for 40 seconds and a touch-down, one-degree decrement for annealing temperature starting with 7°C above *T*_*m*_ for each primer for 30 seconds and 72°C for 1 minute. This was then followed by 30 cycles of 95°C for 40 seconds, a last annealing temperature for 30 seconds and 72°C for 1 minute, and a final extension of 72°C for 10 minutes. The PCR products were separated on 4% metaphor gel with 50 bp DNA ladder (N3231S, Biolabs, Inc., UK). The gel was stained with Midori green, visualized under ultraviolet light, and photographed by ChemilImager Gel Documentation imaging system (Alpha Innotech Corporation, CA, USA).

## 4. Data Analysis

### 4.1. Morphological Data

The recorded data (after normality and homogeneity test) were subjected to analysis of variance (ANOVA) as per two-factor experiment with three irradiation treatments and four varieties arranged in a randomized complete block design (RCBD) with four replications. The analysis was carried out using the portable SAS 9.1 program, and least significant differences (LSD) were used for comparison among treatment means at *P* ≤ 0.05. To evaluate the relationship among the different variables in the experiment, correlation coefficients were used by SAS 9_1_3 portable. To group the individuals based on morphological dissimilarity, cluster analysis was conducted on the Euclidean distance matrix with the unweighted Pair-Group Method using Arithmetic average (UPGMA) using NTSYS software. The same program was used for principal components analysis (PCA) to define eigenvalues and eigenvectors and also for comparison of the mean of groups to define effective traits in separation of the groups. Eigenvectors are the weights in a linear transformation when computing principal component scores while eigenvalues indicate the amount of variance explained by each principal component. The cophenetic correlation coefficient (CCC) was used to measure the goodness of fit of the similarity matrices to their corresponding phenograms in morphological data using PAST (PAleontological Statistics) software V. 2.17 [27].

### 4.2. Molecular Data

Allele size was measured with UVDoc 99.02 analysis software (UVI Tech, Cambridge, UK) by manual editing to increase accuracy. This procedure was carried out two times to exclude wrong scorings. The PowerMarker 3.25 software package [28] was used to produce a dendrogram using UPGMA method. Data were scored as “1” for presence and “0” for absence. The binary data matrix was entered into the Numerical Taxonomy and Multivariate Analysis System (NTSYSpc 2.10e) [29] to generate Dice's similarity matrix. The software POPGENE32, Version 1.32 [29], was used to calculate genetic variation parameters, including observed heterozygosity (the proportion of heterozygous individuals in the population) (*H*_*o*_), expected heterozygosity (*H*_*e*_) [30]—defined as the probability that two randomly chosen alleles from the population are different [31]—observed number of alleles (*n*_*a*_), effective number of alleles (*n*_*e*_), Nei's gene diversity, Shannon's information index (*I*), and percentage of polymorphic loci. To compare the efficiency of primers and polymorphism information content (PIC), a measure of allelic diversity at a locus was calculated using online PIC calculator software (http://www.liv.ac.uk/~kempsj/pic.html) using the following formula:

(1) $$\begin{array}{c} \text{PIC}=1-\overset{n}{\underset{i=1}{\sum }}pi^{2}-\overset{n-1}{\underset{i}{\sum }}\overset{n}{\underset{j=i+1}{\sum }}2pi^{2}pj^{2}, \end{array}$$

where *p*_*i*_ is the frequency of the *i*th allele and *n* is the number of alleles. Markers were classified as informative when PIC was ≥0.5. Principal component analysis (PCA) was also generated for SSR data by NTSYS-pc 2.10e.

## 5. Results and Discussions

### 5.1. Gamma Irradiation and Radiation Sensitivity Test

**Figure 1:**
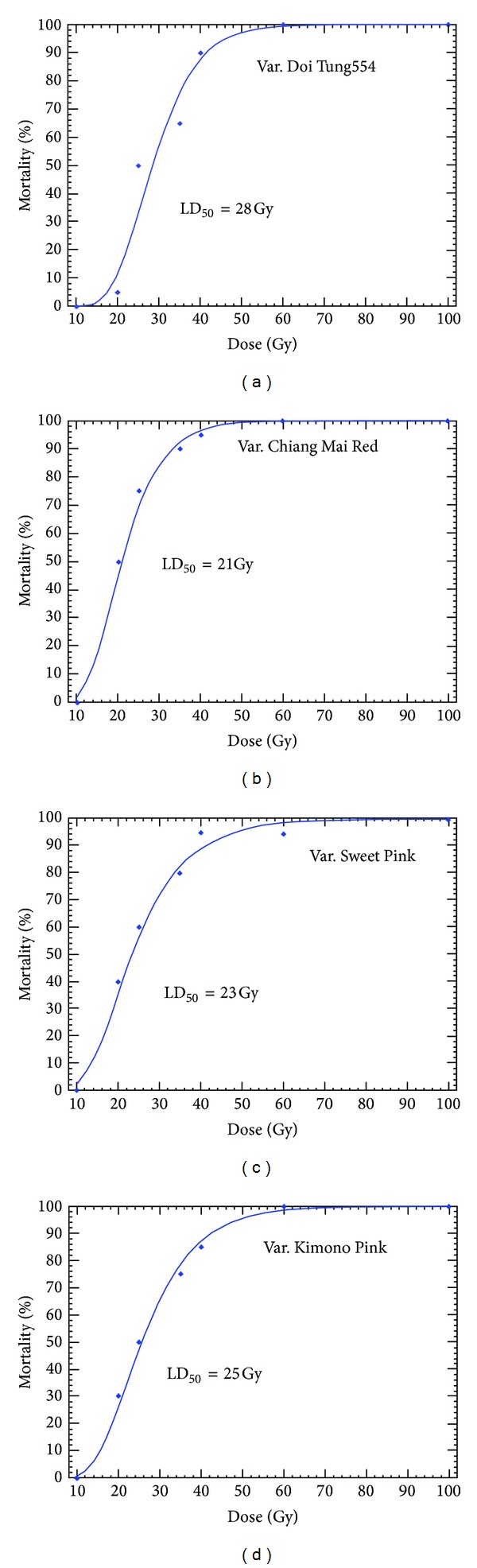
PoloPlus plot of linear scale of dose versus mortality percent. (a) Doi Tung 554, (b) Chiang Mai Red, (c) Sweet Pink, and (d) Kimono Pink.

The sensitivity of *C. alismatifolia* varieties to radiation was evaluated by comparing the mortality rate (%) of irradiated plants at 40 days after irradiation. The plant mortality rate increased with increasing irradiation dosage (Table 3). The hybrid Doi Tung 554 was found to be least sensitive to gamma irradiation than other varieties (51% mortality), while Chiang Mai Red variety showed the lowest survival rate (63% mortality). Sweet Pink and Kimono Pink varieties showed 58% and 55% mortality rate, respectively. At 50% survival rate (LD_50_), the gamma doses administered were 28, 21, 23, and 25 Gy for Doi Tung 554, Chiang Mai Red, Sweet Pink, and Kimono Pink, respectively (Figure 1). Abdullah et al. [32] had previously indicated that the LD_50_ for *C. alismatifolia* var., *Chiang Mai Pink* was approximately at 25 Gy. The death of plants is attributed to the interaction of radiation with other molecules in the cell, particularly water, to produce free radicals (H, OH). The free radicals could combine to form toxic substances, such as hydrogen peroxide (H_2_O_2_), which contribute to the destruction of cells. This indirect effect is especially significant in vegetative cells, the cytoplasm which contains about 80% water [33]. However, sensitivity of the plant material depends on the genetic constitution, dose-employed, DNA amount, moisture content, and stage of development and genotype [34]. The choice of the dose to be applied for the highest mutant rescue is often left to the breeder's experience with the specific plant material, its genetics, and its physiology.

### 5.2. Analysis of Variance (ANOVA) for Morphological Traits of *C. alismatifolia* in M_1_V_1_

Analysis of variance indicated highly significant differences among the varieties, doses, and their interaction for all traits in M_1_V_1_ generation (Table 4). Some desired and undesired abnormalities such as dwarfism, chlorophyll mutation (albinism), striata (yellow or white longitudinal bands altering with green colors), two-midrib leaves, split leaves, double flower stalk in one plant, double inflorescence, marbled pink bracts, two-tone pink-purplish bracts, and two-flag petals were found in M_1_V_1_ plants (Table 5).

### 5.3. Effect of Gamma Irradiation on Vegetative Traits in the M_1_V_1_

The growth of plants treated with 10 and 20 Gy of gamma rays was slower than that of the controls (Table 6). In irradiated plants, the leaf length and leaf width decreased significantly (*P* < 0.05) as the radiation doses increased. This trend is quite common in mutagenised populations. Such effects are known to arise due to drastic chromosomal aberrations in addition to genetic mutations. Similar decreases in leaf size were reported by Pongchawee et al. [35] and Tangpong et al. [36]. These results were in agreement with an earlier study [4] which reported that the growth of chrysanthemum exposed to acute gamma rays was less than the control in the M_1_V_1_ generation. All varieties, doses, and interaction effects resulted in significant differences for number of leaves. Among untreated plants, Kimono Pink variety had higher number of leaves (4.6) than the other three (3, 3, and 3.2) varieties. In Chiang Mai Red and Sweet Pink varieties, plants exposed to 10 Gy showed higher number of leaves than untreated plants. However, at 20 Gy, there was significant reduction in number of leaves for all studied varieties in comparison to control. Similar stimulatory effects were obtained at lower doses in ginger by Hegde [37] and Giridharan and Balakrishnan [38].

Progressive reduction in growth parameters can be interpreted on interference in normal mitosis and frequent occurrence of mitotic aberrations, inhibition of rate of assimilation and consequent change in the nutrient level in the plant, and inactivation of vital enzymes especially those associated with respiration [39]. Dose-dependent negative effect was also detected for plant height. The tallest plants were recorded from the untreated rhizomes (0 Gy) with heights of 111.2, 91.2, 74.6, and 59.1 cm followed by the 10 Gy irradiated plants with heights of 71.4, 54.2, 37.7, and 42.0 cm, and the 20 Gy irradiated plants with corresponding heights of 17.0, 51.2, 19.5, and 28.3 cm for Chiang Mai Red, Doi Tung 554, Sweet Pink, and Kimono Pink varieties, respectively. These results are in agreement with the findings of Abdullah et al. [32]. Reduction in growth parameters and dwarfism can be caused by interference of normal mitosis and frequent occurrence of mitotic aberrations, inhibition of assimilation rates, and consequent changes in nutrient levels in plants. Additionally, mutagenic effects such as auxin destruction, inhibition of auxin synthesis, failure of assimilatory mechanism, and changes in the specific activity of enzymes can cause growth reductions [37]. High doses of ionizing radiation have been shown to damage macromolecular cellular components such as cell walls, membranes, and DNA [40]. The number of shoots also decreased significantly as the radiation doses increased. Radiation also affects organic molecules that are essential to the cell division process, and, thus causing cell division to stop [36].

### 5.4. Effect of Gamma Irradiation on Flowering Development Traits in the M_1_V_1_

All control and 10 Gy irradiated plants produced flowers, while the Chiang Mai Red and Sweet Pink varieties which were exposed to 20 Gy did not go into the flowering stage. Lamseejan et al. [4] also showed that flowering percentage decreases as gamma ray doses are increased. In the present study, gamma rays caused late flowering in all four varieties. Days to appearance of first visible buds were also significantly different among the four varieties (Table 7). Gamma rays caused a noticeable delay in flowering of irradiated plants in comparison to the untreated ones. First visible buds were observed at 65.2 and 87.4 days in the control and 10 Gy treatments, respectively, for the Chiang Mai Red variety. In Doi Tung 554, the first visible buds were appeared at 47.4, 65.6, and 84.0 days after planting at 0, 10, and 20 Gy doses, respectively. In the Sweet Pink variety, the number of days to visible bud appearance increased significantly from 67.8 days in controls to 97.8 days in the 10 Gy irradiated plants. In comparison to other three varieties, the Kimono Pink variety needed the longest time to visible bud appearance and, same as other varieties, there was a positive correlation between the number of days to first visible bud and the gamma irradiation dose. In the control and 10 Gy and 20 Gy irradiated individual plants, flower buds were visible at 85.2, 132.8, and 137.4 days after planting, respectively. Previous studies also showed that onset of flowering and formation of floral parts in mutants of *Arabidopsis thaliana*, maize, barely, pea, and tobacco involved growth regulators (or phytohormones), such as auxins, cytokinins, gibberellins, abscisic acid, ethylene, and brassinosteroids [41]. There were significant differences among treatments for the length of the inflorescence. In all varieties, the longest inflorescence length was observed in the untreated plants with 16.2, 13.4, 14.6, and 13.7 cm lengths for Chiang Mai Red, Doi Tung 554, Sweet Pink, and Kimono Pink, respectively. The corresponding inflorescence lengths were 8.2, 9.4, 7.9, and 10.9 cm for the 10 Gy irradiated plants. The days to anthesis for *C. alismatifolia* varieties were significantly affected by variety, gamma irradiation doses, and their interaction. The number of days to full bloom was noticeable earliest for untreated plants at 74.2, 54.6, 75.6, and 94.2 days for Chiang Mai Red, Doi Tung 554, Sweet Pink, and Kimono Pink varieties, respectively. This was then followed by plants at 10 Gy at 102, 78, 109.8, and 122.4 days. The number of true flowers or the secondary inflorescence developed in the axil of the primary bracts decreased as radiation dosage increased. In the present study, the gamma rays also decreased the days to inflorescence senescence. In this study, there was a strongly, significantly, and positively correlation (0.919**) (data not shown) between the number of true flowers and the number of days to senescence. The number of pink bracts also decreased with increasing radiation dosage. Most gamma ray effects on senescence are considered as resulting from the action of free radicals generated from water and oxygen by the ionizing energy on the cellular components. Membrane deterioration is a general feature of natural senescence and stress-induced aging [42].

Irradiation induced some mutation spectrum of flower color variation that included colors such as purple, pale purple, rather pale purple, white, purple white (marbled pattern), and two-tone purple color.

**Figure 2:**
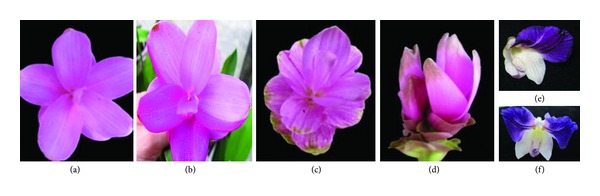
Effect of acute gamma rays on flower traits in *C. alismatifolia*. (a) Untreated inflorescence bracts color, Doi Tung 554. (b) Two tone bract color, Doi Tung 554 (20 Gy). (c) Marbled pattern of bract color, Doi Tung 554 (20 Gy). (d) Double inflorescence within one stalk var. Chiang Mai Red (10 Gy). (e) True flower in nontreated plants. (f) Two-flag petals true flowers (10 Gy).

Mutation spectrum of flower shape variation included double inflorescence within one stalk, double stalk per plant, inflorescence without bracts, two-flag petal true flowers, and chlorophyll mutation in the leaves which are generally caused by induced gamma rays (Figure 2). Ionizing radiation, including gamma rays, induces fragment deletions or insertions that eventually lead to changes in amino acids and a modification of leaf and stem pigmentation [43]. A mutation in the biosynthetic pathway of structural or regulatory genes may cause a change in flower color [44]. When the blockage occurs at the early stages of anthocyanin synthesis, white flowers will result, while a blockage at later stages leads to different flower colors due to the accumulation of particular anthocyanins [45]. Chloroplasts were extremely sensitive to gamma radiation compared to other cell organelles [46].

### 5.5. Effect of Gamma Irradiation on Rhizome Characteristics in Selected Doses in M_1_V_1_

The number of new rhizomes and the number of storage roots per rhizome were significantly affected by varieties, doses, and their interaction (Table 8). The number of new rhizomes only in Kimono Pink variety did not show any differences between untreated and treated plants. The rhizome size was influenced only by doses. As dose level increased, the rhizome size decreased. Among untreated plants, the Sweet Pink rhizomes had the most number of storage roots (7.8) and the Kimono Pink rhizomes had the least number of storage roots (4.0). This was followed by 10 Gy irradiated rhizomes with 4, 4.6, 4.8, and 2.8 storage root numbers for Chiang Mai Red, Doi Tung 554, Sweet Pink, and Kimono Pink, respectively. Irradiation with 20 Gy decreased significantly the number of storage roots in all studied varieties. The plant *C. alismatifolia* is propagated from a propagule (one rhizome + 5-6 t-roots). The rhizome size dose matters in the growth of the plant. Smaller rhizome size usually resulted in smaller plant size with narrow, grass-like leaves. The storage roots play a very important role in the growth and flowering of *Curcuma*. The t-roots make up about 85% of the total fresh weight and 70% of the total dry weight of a typical propagule. The storage organs act for plant growth during dormancy and emergence [47]. More storage root per propagule, resulted in faster flowering and higher yield plant [48]. In this study, also there was significant and negative correlation between number of storage roots and number of days to visible bud appearance (−0.525**).

### 5.6. Cluster Analysis of *C. alismatifolia* for Morphological Traits

**Figure 3:**
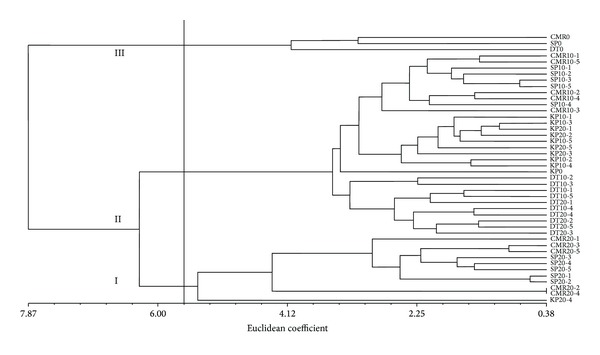
Dendrogram representing the morphological variation among 44 irradiated and nonirradiated individuals of *C. alismatifolia* across 14 variables. CMR = Chiang Mai Red; DT = Doi Tung 554; SP = Sweet Pink; Kp = Kimono Pink.

The morphological data were used to calculate the similarity between the treated and non-treated *C. alismatifolia* samples and UPGMA dendrogram was constructed (Figure 3). The cophenetic correlation coefficient (CCC) value between the genetic dissimilarity matrix estimated from the morphological characters and the UPGMA clustering method was *r* = 0.93, showing a proof fit. This value was higher than of studies on olive for morphological traits (*r* = 0.69) [49]. In this dendrogram, the 44 *C. alismatifolia* samples appeared to form three main clusters and five minor clusters at coefficient level 5.6: cluster I included three non-irradiated members and cluster II included 10 Gy irradiated individual plants of all four varieties and 20 Gy irradiated samples of Doi Tung 554 and Kimono Pink varieties except KP20-4 (no flowering). Main cluster III included the 20 Gy irradiated individuals Chiang Mai Red and Sweet Pink varieties (as mentioned in morphological part, these individuals did not go to flowering stage) and KP20-4. Cluster II can be divided to five subclusters. The first subcluster included 10 members (CMR10-1, CMR10-5, SP10-1, SP10-2, SP10-3, SP10-5, CMR10-2, CMR10-4, SP10-4, and CMR10-3); the second one included nine members (KP10-1, KP10-2, KP10-3, KP10-4, KP10-5KP20-1, KP20-2, KP20-5, and KP20-3). KP0 was the lone member of the third sub-cluster. The fourth sub-cluster included two members only (DT10-2 and DT10-3). The last sub-cluster main cluster II had eight members included (DT10-1, DT10-4, DT10-5, DT20-1, DT20-2, DT20-3, DT20-4, and DT20-5). Mean value of groups for each trait is presented in Table 9. This table clearly shows the different mean values of the three main clusters. The minimum mean value referred to main cluster III while the maximum mean value for number of shoots, leaf length, leaf width, plant height, number of true flowers, inflorescence length, rhizome size, number of new rhizomes, and number of storage roots belonged to cluster I which was included non-treated plants. These results showed that the gamma irradiation has induced morphological changes in *C. alismatifolia* individuals of four studied varieties so that they showed phenotypically differences from their controls.

### 5.7. Principal Component Analysis of *C. alismatifolia* for Morphological Traits

**Figure 4:**
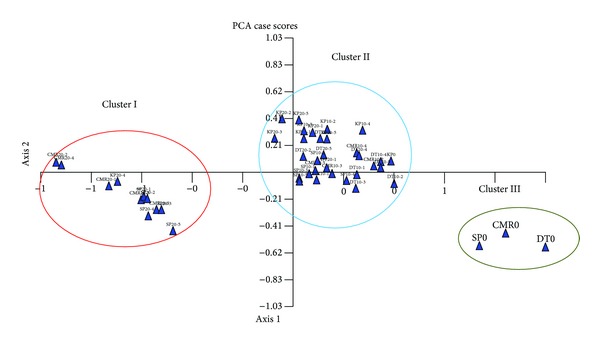
Two-dimensional graph of principal component analysis (PCA) for 14 morphological variables indicating relationships among irradiated and nonirradiated four varieties of *C. alismatifolia.*

To assess the patterns of variation, PCA was done by considering all of the 14 variables. The first three components of PCA explained 82.8% of the total variation (Table 10). Only the first component which accounted for 56.2% of the total variation was attributed to inflorescence length, plant height, days to senescence, number of true flowers, and rhizome size. In PCA three-dimensional graph, the grouping of individuals confirmed the clustering results (Figure 4). The PCA graph proved that all irradiated individual plants of *C. alismatifolia* varieties are phenotypically different from their non-irradiated individuals.

## 6. Molecular Characterization

### 6.1. SSR Polymorphism

Amplifications were successful for all the 17 SSR markers assayed. This reflects a high level of homology between SSR flanking regions in *C. longa* and *C. alismatifolia*. Out of 17 primer pairs, eight primer pairs resulted in polymorphic PCR products.  Table 11 summarizes the results obtained based on the analysis of individuals of the four studied varieties using the polymorphic SSR loci for 0, 10, and 20 Gy irradiated plants. The number of alleles varied widely among these loci. A total of 25, 36, and 41 alleles were observed among 0, 10, and 20 Gy irradiated individual plants, respectively. In the 10 Gy treatment, the number of alleles ranged from three (clon09 and clon14) to seven (clon08) with an average value of 4.5 per locus. In the 20 Gy treatment, the number of alleles varied from three (clon09 and clon14) to seven (clon08 and clon12) with an average value of 5.1 per locus. In the untreated individuals, the number of alleles ranged from two (clon04, clon09, clon11, and clon14) to five (clon12) with an average value of 3.1. The difference between the average number of observed alleles and effective number of alleles was due to the uneven frequency of each allele [50]. For each of the eight SSR primers, PIC values (which measures allele diversity and frequency among varieties) varied from 0.19 (clon04) to 0.71 (clon01) in untreated plants, and from 0.25 (clon04) to 0.75 (clon08) in 10 Gy acutely irradiated plants. In 20 Gy irradiated individual plants, the PIC value ranged from 0.42 (clon14) to 0.75 (clon08 and clon12). The mean PIC for all loci was 0.47, 0.54, and 0.61, for 0, 10, and 20 Gy irradiated plants, respectively.

The PIC value provides an estimate of the discriminatory power of a marker by taking into account not only the number of alleles at a locus but also the relative frequencies of these alleles [51]. Thus, except for clon09 and clon14 which were moderately polymorphic (0.25 < PIC < 0.5), all other loci were highly polymorphic (0.5 < PIC), while none of the loci showed low polymorphism. The average discriminating power of microsatellite markers (0.61) observed in the present study ensures the future utility of microsatellite markers for genetic variation studies in *C. alismatifolia* varieties. Our results reflect similar findings as reported earlier in the study of genetic variation in other species of *Curcuma* using SSR markers [13]. The highest Nei's gene diversity (*h*) was obtained with the 20 Gy treatment (0.61) followed by the 10 Gy treatment (0.6) and the untreated individuals (0.5). The mean Shannon's information index (*I*) was 0.92, 1.14, and 1.30 in the 0, 10, and 20 Gy irradiated plants, respectively. The high value of Shannon's information index represents the effectiveness of microsatellite loci to reveal the variation among these varieties at the different doses used. Overall genetic variability for the varieties studied, represented by the Shannon-Weiner index, was relatively high in comparison to other studies involving *C. longa* accessions [10, 52]. Additionally, the 20 Gy acutely irradiated individuals showed a higher mean percentage of polymorphic loci (63%) than the 10 Gy (58%) and untreated (0.22) ones. This implies that irradiation with a dose of 20 Gy induced more genetic variation in the M_1_V_1_ generation.

### 6.2. Cluster Analysis

**Figure 5:**
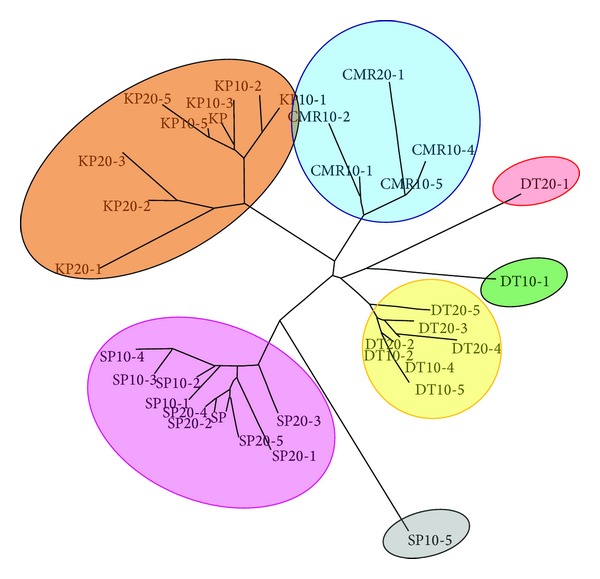
Unrooted neighbor joining tree showing genetic relationship among 44 irradiated and nonirradiated *C. alismatifolia* using SSR markers. The colors of the branches correspond to those of the same cluster.

The dendrogram was constructed using PowerMarker 3.23. The 44 studied individuals were clustered into seven groups (G1–G7). Colors were applied according to our model-based cluster analysis results. Group I is comprised of nine individual plants of Chiang Mai Red variety receiving 0, 10, and 20 Gy treatments (blue color in Figure 5). One individual plant, DT10-1, was assigned to group II (green color in Figure 5). All irradiated and non-irradiated individuals of Doi Tung 554 variety were assigned to group III (yellow color in Figure 5). DT20-1 and SP10-5 were the sole members of groups IV (red color in Figure 5) and V (gray color in Figure 5), respectively. Group VI included treated and untreated individuals of Sweet Pink variety (pink color in Figure 5). Lastly, all Kimono Pink individuals were assigned to cluster VII (orange color in Figure 5). The genetic similarity coefficient (data not shown) among the 44 individuals amplified using eight SSR primers varied from 0.0 to 1.0. The highest value (1.0) corresponded to [(CMR10-3, CMR10-5, CMR, and CMR20-2), (DT10-2 and DT10-3), (DT and DT20-2), (KP20-3, KP-20-4, KP10-3, and KP10-4)] individuals that generated identical fingerprints across the markers studied. Among the studied varieties, the Sweet Pink individual plants showed the lowest similarity (0.0) with Kimono Pink individuals which indicated that they were relatively remote in relationship (Figure 5). An overview of the clustering pattern indicates that the grouping of the studied individual plants was based on different varieties and largely independent of the doses of gamma irradiation. A wide range in similarity values had also been observed in different species of *Curcuma* [12, 13, 52, 53].

### 6.3. Principle Component Analysis (PCA)

**Figure 6:**
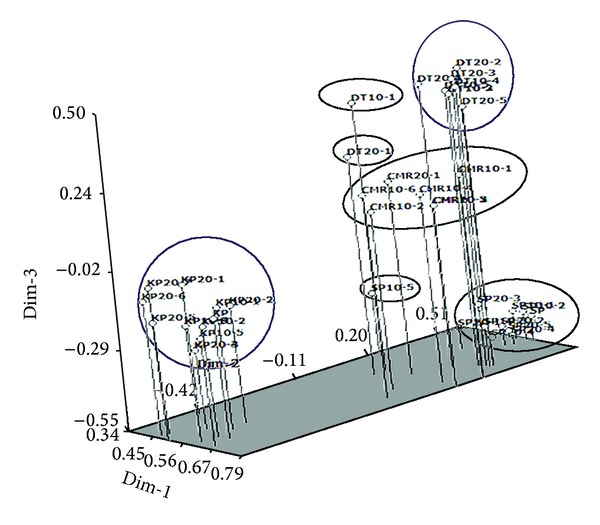
Three-dimensional graph of principal component analysis (PCA) indicating relationships among irradiated and nonirradiated four varieties of *C. alismatifolia.*

The data generated from 44 *C. alismatifolia* individual plants were subjected to principal component analysis (PCA) to visualize individuals in a multivariate space. In the three-dimensional graph derived from the SSR analysis, all studied individuals were grouped into seven clusters (Figure 6). In PCA, the first three principal components (PC) extracted a cumulative of 68.26% of the total variation among the 44 individuals of *C. alismatifolia.* The first three coordinates, PC1, PC2, and PC3, accounted for 41.36%, 15.78%, 11.12% of the total variation, respectively. The distribution of the individual plants in a three-dimensional plot using the first three principal components showed the genetic relationship between individual plants. It was evident that both methods, dendrogram and three-dimensional plots of PCA, were effective in illustrating genetic relationships and the groups found were comparable [11, 13]. The PCA results were similar to those obtained from cluster analysis, where all individuals from different varieties were assigned to different groups. These graphical illustrations enable the assessment of the relationship/distances among all of the individuals in the study [50].

## 7. Conclusion

This is the first attempt to evaluate the effect of acute gamma irradiation on *C. alismatifolia* varieties using both morphological characteristics and molecular markers. In plant breeding programs, mutagenic treatments with low negative physiological effects and strong genetic effects are desirable. Hence, we used more effective doses of gamma irradiation (10 and 20 Gy) of which particularly 20 Gy dose was effective to influence morphological and molecular characteristics of studied *Curcuma alismatifolia* varieties. The lower dose (10 Gy) of radiation probably caused little damage to the plants genetic material so that the cells could repair themselves in next generation. As a result, in this study, 20 Gy acute gamma irradiation resulted in a higher percentage of mutation and getting desired mutants was more possible. Based on the LD_50_ values determined in this study, it was apparent that the varieties Chiang Mai Red and Sweet Pink were more sensitive to gamma rays compared to Doi Tung 554 and Kimono Pink. Our results show that the variety Doi Tung 554 (*Curcuma* hybrid) had the most morphological responses to gamma rays. The use of microsatellite markers as a codominant marker will facilitate the exploration of genetic variability among treated and non-treated plants and will help to distinguish the plants showing differences in morphological characters. The overall effects on the M_1_V_1_ generation revealed that acute gamma irradiation at optimum doses has the potential for developing new varieties of *C. alismatifolia *with improved commercial properties suitable for the Malaysian flower industry.

## Figures and Tables

**Table 1:** Important discriminating qualitative features of studied *C*. *alismatifolia* varieties and hybrid varieties for M_1_V_1_ generation.

Variety	Floral characters						Leaf characters					
	Plant type	Spike position	Color of calyx	Color of corolla	Inflorescence lower bract color	Inflorescence coma bract color	Leaf habit	Color of leaf sheath	Leaf margin	Leaf color	Leaf midrib color	Leaf shape
Chiang Mai Red	Erect	Terminal	White	Light Purple-white	Green	Purple N78A*	Erect	Purple-green	Smooth	Dark green	Purple	Long, narrow, and stiff
Doi Tung 554	Erect	Terminal	White	Dark Purple-white	2014	Red purple N78C*	Erect	Dark Purple	Smooth	Dark green	Purple	Long, narrow, and stiff
Sweet Pink	Erect	Terminal	White	Dark Purple-white	Green	Purple-violet N80D*	Erect	Purple-green	Low wavy	Light green	Purple	Wide and stiff
Kimono Pink	Erect	Terminal	White	Dark Purple-white	Green	Purple-violet N80C*	Erect	Purple-green	Medium wavy	Dark green	Green	Long and narrow

*The Royal Horticulture Society (RHS) London color chart.

**Table 2:** List of morphological traits and brief descriptions.

Number	Morphological traits
1	Number of new shoots (number): total number of produced new shoots per rhizome
2	Leaf length (cm): length of the fully opened first leaf from the soil surface to leaf tip
3	Leaf width (cm): breadth of the leaf at the widest part of the leaf
4	Number of leaves (number): number of fully emerged leaves at the end of vegetative growth stage
5	Plant height (cm): the height of the peduncle at the top of the soil surface to the tip of the inflorescence
6	Number of days to visible bud appearance (days): number of days from the first day of planting to appearance of the first visible bud. Buds appear at the middle of two sheaths of leaves
4	Inflorescence length (cm): the length between the lowest green bracts to tip of the upper pink bracts during anthesis
8	Number of days to anthesis (days): the number of days from planting to fully opened flower bud
9	Number of days to senescence (days): the days from first day of anthesis until end of the shelf life of the flower
10	Number of true flowers (No.): the number of small axillary flower buds which develop inside bracts during anthesis
11	Number of pink bracts (No.): inflorescence of *C. alismatifolia* comprising several apical bracts. Most basal bracts are green, but the distal ones, more numerous than the green ones, are purplish pink bracts which determine the attractiveness of the flowering stems. The number of pink bracts was counted during anthesis
12	Rhizome size (cm): the girth of the M_0_V_0_ and M_1_V_1 _rhizomes was measured with vernier caliper and mean was expressed in centimeter
13	Number of new rhizomes (No.): after harvesting, the total number of new rhizomes (M_1_V_1_) was recorded
14	Number of storage roots (No.): the total number of storage roots of M_0_V_0_ and M_1_V_1_ rhizomes was recorded at harvesting time

**Table 3:** The number of irradiated and mortal rhizomes of *Curcuma alismatifolia* varieties after acute irradiation with different doses of gamma rays.

Dose (Gray)	Total number of irradiated rhizomes in each var.	Number of mortal rhizomes after 40 days				Mortality rate (%) in each dose
		Doi Tung 554	Chiang Mai Red	Sweet Pink	Kimono Pink	
0	20	0	0	0	0	0
10	20	0	0	0	0	0
20	20	1	10	8	6	31.2
25	20	10	15	12	10	58.7
35	20	13	18	16	15	77.5
40	20	18	19	19	17	91.2
60	20	20	20	19	20	98.7
100	20	20	20	20	20	100
Total	**160**	**81**	**102**	**94**	**88**	
Mortality rate (%)		51	63	58	55	
LD_50_ (%)		28 Gy	21 Gy	23 Gy	25 Gy	
Confidence limits (95%)		25–31 Gy	17–23 Gy	19–26 Gy	22–28 Gy	

**Table:** (a)

Source of variation	Mean squares							
	df	Generation	Number of shoot	Leaf length	Leaf width	Leaf number	Days to visible bud	Plant height
Block	4	M_1_V_1_	0.05	62.2	1.1	0.14	94.67	83.58
Variety	3	M_1_V_1_	2.63*	270.2*	30.11*	5.00*	14556.02*	2537.39*
Dose	2	M_1_V_1_	6.11*	3738.04*	41.37*	10.42*	10120.01*	15707.66*
Var.*dose	6	M_1_V_1_	1.41*	85.55*	4.18*	1.67*	7312.71*	1159.34*
Error	44	M_1_V_1_	0.094	29.51	0.77	0.37	49.03	
Total	**59**							**84.93**

CV (%)		M_1_V_1_	23	18.3	17.6	20.6	9.1	16.5

**Table:** (b)

Source of variation	Mean squares									
	df	Generation	Days to anthesis	Days to senescence	No. of true flow	No. of pink bract	Inflorescence length	No. of new rhizomes	Rhizome size	No. of storage roots
Block	4	M_1_V_1_	128.47	0.48	4.18	0.72	2.82	0.1	0.18	0.55
Variety	3	M_1_V_1_	16080.17	100.91*	91.66*	625.43*	37.6*	1.63*	0.23^ns^	17.26*
Dose	2	M_1_V_1_	15872.61	1286.82*	582.86*	353.15*	560.22*	10.31*	5.31*	64.81*
Var.*dose	6	M_1_V_1_	8769.57	34.64*	29.00*	9.77*	40.04*	1.28*	0.25ns	3.32*
Error	44	M_1_V_1_	77.52	3.22	3.92	2.26	4.45	0.11	0.11	1.21
Total	**59**									

CV (%)		M_1_V_1_	10	15	22.9	16.9	16.5	23	18	26.8

*Significant with least square difference test, *P* < 0.05.

**Table 5:** Effect of acute gamma rays on vegetative and flowering traits of *C. alismatifolia* in the M_1_V_1_ generation.

Variety irradiated dose	Observed variations	Flower color variation
Chiang Mai Red		
10	Dwarfism, no pink bracts inflorescence, small inflorescence, two-flag petal true flower, double inflorescence, undulate leaf margin, and yellow/white strip leaves	Light purple, N78C*
20	Dwarfism, narrow small leaves, and yellow/white strip leaves	No flower
Doi Tung 554		
10	Dwarfism, two-midrib leaves, white/yellow strip leave	Two tone-pink bracts, N74B, N74D*
20	Dwarfism, and white/yellow strip leave	Marble pattern of bracts
Sweet Pink		
10	Dwarfism, two-flag petal true flower, small inflorescence	White bracts/light purple, 75B*
20	Dwarfism, and narrow small leaves	No flower
Kimono Pink		
10	Dwarfism, fewer pink bracts	Light purple, N80D*
20	Dwarfism, fewer pink bracts, and yellow/white strip leaves. Small and narrow leave	Light purple, N80D*

*Royal British Society color chart (RHS).

**Table 6:** Effect of acute gamma rays on vegetative traits of *C. alismatifolia* in M_1_V_1_ generation.

Dose (Gray)	Shoot number	Leaf number	Leaf length (cm)	Leaf width (cm)	Plant height (cm)
CMR					
0	2.0 ± 0.0^a^	3.0 ± 0.4^a^	55.6 ± 0.8^a^	7.1 ± 0.5^a^	111.2 ± 2.5^a^
10	1.0 ± 0.0^b^	3.6 ± 0.5^a^	27.2 ± 2.5^b^	4.5 ± 0.5^b^	71.4 ± 9.6^b^
20	1.0 ± 0.0^b^	1.4 ± 0.5^b^	12.4 ± 12.0^c^	2.5 ± 2.4^c^	17.0 ± 8.0^c^
DT					
0	3.0 ± 0.0^a^	3.0 ± 0.0^a^	62.2 ± 0.8^a^	6.5 ± 0.5^a^	91.2 ± 1.9^a^
10	1.6 ± 0.5^b^	2.8 ± 0.4^a^	28.0 ± 4.4^b^	4.6 ± 0.6^b^	54.2 ± 7.6^b^
20	1.2 ± 0.4^b^	2.4 ± 0.5^a^	24.2 ± 3.8^b^	3.8 ± 0.5^c^	51.2 ± 13.8^b^
SP					
0	1.6 ± 0.5^a^	3.2 ± 0.4^a^	46.1 ± 2.7^a^	10.1 ± 0.4^a^	74.6 ± 3.8^a^
10	1.0 ± 0.0^b^	3.6 ± 0.5^a^	27.5 ± 3.4^b^	5.7 ± 0.2^b^	37.7 ± 13.7^b^
20	1.0 ± 0.0^b^	2.0 ± 1.0^b^	19.6 ± 7.4^c^	5.7 ± 0.4^b^	19.5 ± 7.3^c^
KP					
0	1.4 ± 0.0^a^	4.6 ± 0.5^a^	34.5 ± 2.9^a^	4.7 ± 0.4^a^	59.1 ± 2.8^a^
10	1.0 + 0.5^a^	4.0 ± 0.5^b^	22.0 ± 2.4^b^	3.1 ± 0.4^b^	42.0 ± 5.4^b^
20	1.0 ± 0.0^a^	3.2 ± 0.4^b^	15.6 ± 4.3^c^	3.1 ± 1.1^b^	28.3 ± 17.4^c^

CV (%)	23	18	26	16	16.5

Means with the same or common letter are not significantly different; least square difference test, *P* < 0.05.

**Table 7:** Effect of acute gamma rays on flower development characteristics of *C. alismatifolia* in M_1_V_1_ generation.

Dose (Gy)	Days to visible bud	Inflorescence length (cm)	Days to anthesis	Number of true flowers	Number of Pink bracts	Days to senescence
CMR						
0	65.2 ± 4.7^b^	16.2 ± 0.57^a^	74.2 ± 5.4^b^	13.2 ± 1.9^a^	10.4 ± 1.5^a^	21 ± 1.0^a^
10	87.4 ± 9.5^a^	8.2 ± 3.97^b^	102.0 ± 7.1^a^	5.6 ± 1.9^b^	5.0 ± 0.7^b^	10 ± 1.4^b^
20	0.0 ± 0.0^c^	0.0 ± 0.0^c^	0.0 ± 0.0^c^	0.0 ± 0.0^c^	0.0 ± 0.0^c^	0.0 ± 0.0^c^
DT						
0	47.4 ± 4.3^c^	13.4 ± 0.54^a^	54.6 ± 5.4^c^	16.2 ± 1.3^a^	23.2 ± 0.4^a^	23 ± 0.0^a^
10	65.6 ± 3.7^b^	9.4 ± 1.9^b^	78.0 ± 3.7^b^	11.2 ± 2.1^b^	16.6 ± 0.8^b^	12.2 ± 1.30^b^
20	84.0 ± 4.2^a^	7.6 ± 0.82^c^	99.4 ± 4.9^a^	8.8 ± 1.3^b^	15.6 ± 1.1^b^	10.6 ± 1.8^b^
SP						
0	67.8 ± 2.1^b^	14.6 ± 0.89^a^	75.6 ± 2.6^b^	16.2 ± 1.3^a^	10.0 ± 2.4^a^	18.8 ± 0.83^a^
10	97.8 ± 18.1^a^	7.9 ± 2.0^b^	109.8 ± 18.2^a^	6.8 ± 2.5^b^	5.2 ± 0.8^b^	9.2 ± 2.4^b^
20	0.0 ± 0.0^c^	0.0 ± 0.0^c^	0.0 ± 0.0^c^	0.0 ± 0.0^c^	0.0 ± 0.0^c^	0.0 ± 0.0^c^
KP						
0	85.2 ± 2.3^c^	13.7 ± 0.44^a^	94.2 ± 1.9^c^	11.2 ± 1.2^a^	9.6 ± 2.1^a^	18 ± 1.8^a^
10	104.4 ± 6.5^b^	10.8 ± 1.08^ab^	122.4 ± 10.0^b^	7.6 ± 2.7^b^	6.4 ± 1.6^ab^	10.2 ± 1.7^b^
20	127.2 ± 6.5^a^	7.90 ± 4.9^b^	142.6 ± 5.8^a^	6.0 ± 3.8^b^	4.2 ± 2.4^b^	6.6 ± 3.7^b^

CV (%)	10	23	9	22	16.9	15

Means with the same or common letter are not significantly different; least square difference test, *P* < 0.05.

**Table 8:** Effect of acute gamma rays on rhizome characteristics of *C. alismatifolia* in M_1_V_1_ generation.

Dose (Gray)	Rhizome size	No. of new rhizomes	No. of storage root
CMR			
0	2.3 ± 0.2^a^	2.6 ± 0.55^a^	4.6 ± 0.54^a^
10	1.8 ± 0.05^a^	1.2 ± 0.44^b^	4.0 ± 0.7^a^
20	0.8 ± 0.7^b^	0.6 ± 0.54^b^	1.4 ± 1.3^b^
DT			
0	2.2 ± 0.1^a^	3.0 ± 0.0^a^	7.2 ± 1.09^a^
10	1.8 ± 0.07^b^	1.4 ± 0.54^b^	4.6 ± 1.3^b^
20	1.6 ± 0.05^b^	1.0 ± 0.0^b^	2.8 ± 1.3^c^
SP			
0	2.2 ± 0.2^a^	2.4 ± 0.54^a^	7.8 ± 2.1^a^
10	1.7 ± 0.07^b^	1.0 ± 0.0^b^	4.8 ± 0.44^b^
20	1.2 ± 0.2^c^	1.0 ± 0.0^b^	2.8 ± 0.83^b^
KP			
0	2.1 ± 0.05^a^	1.0 ± 0.0^a^	4.0 ± 0.0^a^
10	1.6 ± 0.1^ab^	1.0 ± 0.0^a^	2.8 ± 0.44^b^
20	1.1 ± 0.6^b^	1.0 ± 0.0^a^	2.2 ± 0.83^b^

CV (%)	18	23	26

Means with the same or common letter are not significantly different; least square difference test, *P* < 0.05.

**Table 9:** Cluster means for 14 characters estimated in 44 individuals of *C. alismatifolia*.

Clusters	Shoot number	Leaf length	Leaf width	Leaf number	Visible bud (days)	Inflorescence length	Plant height	Anthesis (days)	Senescence (days)	True flow number	Pink bract number	Rhizome size	New rhizome number	Storage root number
I	2.20	54.64	7.36	3.00	60.13	14.73	92.33	69.26	20.93	15.2	14.53	2.29	2.66	6.53
II_1_	1.00	27.35	5.12	3.60	92.60	8.09	54.57	105.9	9.60	6.20	5.10	1.77	1.10	4.40
II_2_	1.22	19.22	2.98	3.44	114.22	10.43	39.10	131.22	9.33	7.55	5.88	1.56	1.00	2.66
II_3_	1.00	34.52	4.16	4.60	85.20	13.70	59.10	94.20	18.0	12.0	9.60	2.06	1.00	4.00
II_4_	1.50	25.00	4.5	2.50	65.00	9.25	49.50	77.00	11.50	9.50	17.00	1.85	2.00	6.00
II_5_	1.37	26.37	4.17	2.62	77.25	8.40	53.56	91.62	11.37	10.12	15.87	1.70	1.00	3.12
III	0.54	15.00	4.65	1.81	0.00	0.00	0.00	0.00	0.00	0.00	0.00	0.90	0.81	2.00

**Table 10:** Eigenvectors, eigenvalues, and proportions of variability for three principle components among 14 characters for 44 *C. alismatifolia*.

Variable	PC1	PC2	PC3
Number of new shoot	0.270	−0.076	−0.173
Leaf length	0.269	−0.305	0.033
Leaf width	0.096	−0.455	0.430
Leaf number	0.210	0.285	0.490
Days to visible bud	0.199	0.462	0.181
Plant height	*0.321 *	0.027	−0.071
Inflorescence length	*0.318 *	0.166	−0.040
Days to anthesis	0.205	0.460	0.148
Days to senescence	*0.342 *	0.017	−0.131
Number of true flower	*0.323 *	0.029	−0.238
Number of pink bracts	0.266	−0.028	−0.546
Rhizome size	*0.302 *	−0.101	0.147
Number of new rhizome	0.250	−0.293	0.028
Number of storage roots	0.264	−0.247	0.298
Eigenvalue	**7.877**	**2.694**	**1.032**
Proportion	**56.264**	**19.246**	**7.371**
Cumulative	**56.264**	**75.511**	**82.882**

**Table 11:** SSR primers, number of alleles, product size, expected heterozygosity, observed heterozygosity, number of effective alleles, Nei's gene diversity, Shannon's Index, and PIC.

Locus	Primer sequence (5′–3′)	Repeat motif	Dose (Gy)	*N*_*a*_	Range size (bp)	*T*_*m*_ (°C)	*T*_*a*_ (°C)	*H*_*e*_	*H*_*o*_	Ne	*h*	*I*	PIC
Clon01	F: ACTGGACTGTCCGAGAGCAT R: TCGTTTAGCGACAACGGATT	(TA)_6_TTG(TC)_16_	0	4	194–212	60.7	68–58	0.78	0.00	4.00	0.75	1.38	0.71
			10	5	194–212	60.7	68–58	0.78	0.00	4.25	0.76	1.51	0.72
			20	6	194–216	60.7	68–58	0.81	0.04	4.80	0.78	1.64	0.73

Clon04	F: TAAATTTGCGAAGGCAATCC R: CCGCAGAGGAATTTGAAGAG	(TATAG)_2_(AG)_17_	0	2	179–200	58.3	65–55	0.22	0.25	1.28	0.21	0.37	0.19
			10	3	179–200	58.3	65–55	0.28	0.24	1.38	0.27	0.52	0.25
			20	6	170–200	58.3	65–55	0.58	0.21	2.31	0.56	1.21	0.54

Clon08	F: CCGGTGAGGGTGATATCTTG R: AAGCTCAAGCTCAAGCCAAT	(GT)_10_	0	4	245–274	60.7	68–58	0.67	0.75	2.91	0.65	1.21	0.61
			10	7	231–274	60.7	68–58	0.79	0.56	4.57	0.78	1.67	0.75
			20	7	245–274	60.7	68–58	0.79	0.39	4.42	0.78	1.65	0.75

Clon09	F: GGAGGAGGCAGTTGATTTGT R: GCTTTGGTGGCTAGAGATGC	(AC)_14_	0	2	182–188	60.4	67–57	0.38	0.00	1.60	0.37	0.56	0.31
			10	3	182–200	60.4	67–57	0.40	0.04	1.65	0.39	0.64	0.33
			20	3	182–195	60.4	67–57	0.51	0.00	1.99	0.50	0.84	0.43

Clon10	F: GTGGGAATTGGATTGCTCTC R: GAGAACTCCCCATGCTTCAG	(GT)_7_	0	4	204–232	60.7	68–58	0.67	0.25	2.91	0.65	1.21	0.61
			10	5	200–232	60.7	68–58	0.66	0.20	2.86	0.65	1.27	0.61
			20	6	200–232	60.7	68–58	0.71	0.26	3.38	0.71	1.40	0.65

Clon11	F: GGGCTTTGTTTAGTTGTCGTG R: CAGGAATGAAGTCGGCAAC	(AGA)_8_	0	2	160–167	60.7	68–58	0.51	0.00	2.00	0.50	0.69	0.37
			10	4	163–182	60.7	68–58	0.64	0.00	2.72	0.63	1.13	0.56
			20	5	163–182	60.7	68–58	0.62	0.00	2.58	0.61	1.12	0.54

Clon12	F: GATTGGATCACATGGTGTGC R: TGGGTTGATGGTTTCTCTGTT	(CT)_20_	0	5	195–231	59.0	66–76	0.71	0.50	3.20	0.68	1.38	0.65
			10	6	195–231	59.0	66–76	0.73	0.28	3.52	0.71	1.45	0.67
			20	7	195–255	59.0	66–76	0.78	0.56	4.39	0.77	1.70	0.75

Clon14	F: TCAGTCGAGGGGTTCCTACT R: GAGAGCTGATCGCAAAAACC	(CTT)_7_	0	2	175–181	60.7	68–58	0.38	0.00	1.60	0.37	0.57	0.31
			10	3	171–181	60.7	68–58	0.55	0.00	2.21	0.54	0.91	0.47
			20	3	171–181	60.7	68–58	0.51	0.00	2.1	0.51	0.81	0.42

Total			0	25									
			10	36									
			20	41									

Mean			0	3.1				0.5 ± 0.1	0.2 ± 0.2	2.5 ± 0.9	0.52 ± 0.1	0.9 ± 0.4	0.47
			10	4.5				0.6 ± 0.1	0.1 ± 0.1	2.9 ± 1.1	0.60 ± 0.1	1.1 ± 0.4	0.54
			20	5.1				0.6 ± 0.1	0.2 ± 0.2	2.9 ± 1.2	0.61 ± 0.1	1.2 ± 0.4	0.61

na: number of alleles *H*_*o*_: observed heterozygosity; *H*_*e*_: expected heterozygosity; Ne: effective number of alleles; *h*: Nei's gene diversity; *I*: Shannon's index; PIC: polymorphic information content.
